# WHY DO I CARE? MOTIVATIONS OF SURVIVORS OF PARENTAL CHILDHOOD HARM WHO ARE CARING FOR THEIR PARENTAL PERPETRATOR

**DOI:** 10.1093/geroni/igad104.0959

**Published:** 2023-12-21

**Authors:** Jaime Goldberg, Jooyoung Kong

**Affiliations:** University of Wisconsin-Madison , Madison, Wisconsin, United States; University of Wisconsin-Madison, Madison, Wisconsin, United States

## Abstract

Adult child survivors of parental childhood maltreatment may remain in relationship with their parental perpetrator across the life course and even take on the caregiving role when their parent becomes older and/or ill. Yet, becoming a caregiver for a parent who was harmful is associated with increased depressive symptoms, heightened stress responses, and maladaptive coping for the caregiver. Given that survivors of parental childhood maltreatment may find caregiving to be detrimental to their mental health and well-being, it is crucial to better understand why an adult child would choose to engage in caregiving for their parental perpetrator. This qualitative research, grounded in Intergenerational Solidarity Theory, examines the motivations to participate in parental caregiving through serious illness or the end of life for survivors of childhood parental harm. Based on the phenomenological perspective, in-depth semi-structured interviews were conducted with 22 adults ages 24-70. Participants were recruited through word-of-mouth and social media. Content analysis revealed five themes: 1) obligation and ambivalence; 2) differentiating from the abuser; 3) “a second chance;” 4) belief systems that influence caregiving; and 5) financial incentives. This information heightens awareness of the experiences of this often-overlooked population of caregivers and invites gerontological researchers and practitioners to consider the complexity in their decision to participate in their perpetrator’s care. Gaining a better understanding of the lived experiences of adult children who are caregivers for their parental perpetrator and their motivations for participating in such care can inform the development of trauma-informed assessment and intervention strategies tailored to this population.

